# The COVID-19 Pandemic as a Natural Experiment: Advancing the Understanding of Stress, Rhythm Dysregulation, and Mental Health in Healthcare Workers

**DOI:** 10.2174/0117450179512031261020111641

**Published:** 2026-10-27

**Authors:** Noemi Maria Mereu, Roberto Demontis

**Affiliations:** 1 PhD course on Capacity Building for Global Health, Department of Medical Sciences and Public Health, University of Cagliari, Cagliari, Italy; 2 Department of Medical Sciences and Public Health, University of Cagliari, Cagliari, Italy

**Keywords:** COVID-19, Healthcare workers, DYMERS, Rhythm dysregulation, Stress, Mental health, Burnout, Transdiagnostic

## Abstract

Few events in recent history have forced clinicians and researchers to rethink mental health quite like COVID-19. In this commentary, we reflect on something that struck us while reading the work of Cantone *et al.*: the pandemic, for all its devastation, created a kind of unplanned observational window, a chance to study, at population scale, what happens when stress and rhythm disruption hit at the same time. Healthcare Workers (HCWs) were at the epicentre of this. They didn't just witness the crisis; they absorbed it, shift after shift, for months. What the data show is that their depressive symptoms and rhythm instability were not random; they appear systematically linked, and at levels one would more typically associate with mood disorder research. We discuss these findings through the lens of DYMERS (Dysregulation of Mood, Energy, and Social Rhythms Syndrome), a heuristic transdiagnostic construct that, in our view, captures something that standard categorical diagnoses tend to miss: the subthreshold, fluctuating psychopathology that sits just below the threshold of formal diagnosis but causes real impairment. The pandemic may not have created these vulnerabilities, but it may well have sharpened our ability to see them.

## 1 INTRODUCTION

The study by Cantone *et al*. [1] comes at a time when the debate on the psychological impact of the pandemic on healthcare workers (HCWs) is still very much open, and offers, in our view, a clinically relevant contribution. Beyond the empirical data, the work invites broader reflection. The pandemic, after all, can be read as an unprecedented natural experiment: millions of people exposed, at the same moment, to intense stress and the destabilization of their biological and social rhythms. We use the term 'natural experiment' loosely here; the pandemic obviously lacked the control conditions of a designed study, and the observational data it generated are subject to the usual limitations: confounding variables, selection bias, and the impossibility of drawing causal conclusions. What it offered, rather, was an unusually clear window onto processes that are difficult to study under controlled conditions.

No controlled study could have replicated what the pandemic did in reality: it altered sleep, social relationships, work, and the capacity to regulate emotions, all at once. The lockdowns, the organizational chaos, and the uncertainty that dragged on for months- all of this ended up destabilizing circadian and social rhythms on a global scale. HCWs found themselves at the centre of all this, chronically exposed to both stress and rhythm instability. This is precisely why they represent a valuable population for understanding how these mechanisms translate into psychopathological vulnerability.

The data from Cantone *et al.* are, notably, striking: the levels of rhythm dysregulation detected in HCWs approach -though do not equal- those reported in some bipolar disorder samples in the same geographic area [1]. This finding should be interpreted with caution, given the cross-sectional design and the limitations of self-reported measures, but it nonetheless raises important questions about the clinical significance of rhythm instability in non-psychiatric populations. Taken together, these findings fit coherently within the Social Zeitgeber framework [2], reinforcing the idea that rhythm disruption is not an epiphenomenon but a core mechanism in mood vulnerability.

It is within this context that the construct of Dysregulation of Mood, Energy, and Social Rhythms Syndrome (DYMERS) offers a potentially valuable transdiagnostic perspective. However, it should be noted that this remains a heuristic hypothesis rather than a fully validated diagnostic category. Initially proposed as a heuristic hypothesis to explain MDQ positivity beyond the diagnosis of bipolar disorder [3, 4], DYMERS tentatively describes a cluster of features - mood instability, altered energy regulation, and disruption of biological and social rhythms - whose clinical relevance is supported by preliminary evidence but has yet to be confirmed through rigorous validation studies. Individuals with these features may experience significant impairment in quality of life even in the absence of formal psychiatric diagnoses [4]. Growing evidence suggests that circadian disruption may represent a transdiagnostic vulnerability factor across a range of neuropsychiatric conditions [5]. A conceptual representation of this framework is provided in Fig. (**1**).

Subsequent research has progressively broadened the picture. MDQ positivity, for example, has been associated with rhythm dysregulation and worsening quality of life across very different settings, not only in psychiatric populations, but also in those without any diagnosis [4, 6]. A particularly interesting finding comes from chronic illness: in systemic lupus erythematosus, rates of MDQ positivity are elevated, suggesting that these instruments may be identifying something broader than bipolar disorder, perhaps a stress-related syndrome with rhythm dysregulation as the common thread [7].

The pandemic, in some sense, put this hypothesis to the test on a large scale. In HCWs subjected to prolonged stress, the hallmark features of DYMERS are found with notable frequency: subthreshold symptoms, hyperactivation, destabilized rhythms. One aspect is particularly notable: suicidal ideation and death-related thoughts were documented even in individuals who did not present a full depressive episode [8]. This raises important questions about the real clinical weight of subthreshold conditions. On the burnout front, DYMERS has been tentatively proposed as a possible underlying mechanism, not mere exhaustion, but a chronic stress syndrome in which the dysregulation of biological and social rhythms may play a key role [6]. This remains, however, an area where further empirical work is needed. Independent systematic reviews have documented that burnout affected nearly half of healthcare workers during the pandemic [9, 10], underscoring the scope of the problem.

DYMERS has also been hypothesized as a possible shared vulnerability substrate across multiple psychiatric conditions, though this remains speculative. Preliminary findings suggest that rhythm dysregulation and hyperactivation may play a role not only in mood disorders but also in panic disorder, where elevated rhythm instability has been observed independently of depressive episodes [11], a finding that, if replicated, would lend further support to a transdiagnostic reading of this construct.

It is worth acknowledging that DYMERS is not the only framework available for understanding stress-related psychopathology in this population. The stress-diathesis model, allostatic load theory [12], and circadian rhythm-based models [5, 13] all offer complementary perspectives on how chronic stress translates into psychiatric vulnerability. Rather than positioning DYMERS as a replacement for these frameworks, we suggest it may function as an integrative lens, one that is particularly attentive to the rhythmic dimension of psychopathology and that may capture features these other models address less directly.

In this sense, the pandemic offered something rare: the opportunity to observe stress and rhythm dysregulation amplifying simultaneously at a population scale, under real-world conditions. HCWs, by virtue of their extreme exposure, represent in this framework a kind of *sentinel population*: it is in them that these mechanisms manifest with the greatest clarity.

It should also be said that the effects did not end with the acute phase. In the post-pandemic period, organizational stress, job dissatisfaction, and systemic difficulties that show no sign of resolving continue to be documented [14]. The “natural experiment” is not over: its consequences extend over time, making a longitudinal, prevention-oriented perspective all the more urgent.

The psychoeducational intervention proposed by Cantone *et al*. fits well within this framework [1]. Working on the regularity of sleep, daily routines, and social interactions means intervening on precisely the components that DYMERS identifies as critical. Preventive strategies based on rhythm regulation may therefore represent a promising avenue for reducing vulnerability and enhancing resilience.

## CONCLUSION

In conclusion, the study by Cantone *et al.* contributes not only to the understanding of HCWs' mental health in the post-pandemic context but also to the broader evolution of psychopathological models. Conceptualizing the COVID-19 pandemic as a natural experiment - with all the methodological caveats this entails - highlights the potential value of integrative frameworks such as DYMERS in capturing the dynamic interplay between stress and rhythm dysregulation. It bears repeating that DYMERS remains a hypothesis-generating construct, and its empirical validation across clinical populations is still needed. Several limitations inherent to interpreting findings from previously published observational studies should also be acknowledged: the cross-sectional designs, reliance on self-report, and the absence of longitudinal follow-up all constrain the conclusions that can be drawn. Future research should focus on operationalizing this construct, testing it across diverse populations, and evaluating targeted interventions aimed at restoring rhythm stability and preventing the progression to more severe psychiatric conditions.

## Figures and Tables

**Fig. (1):**
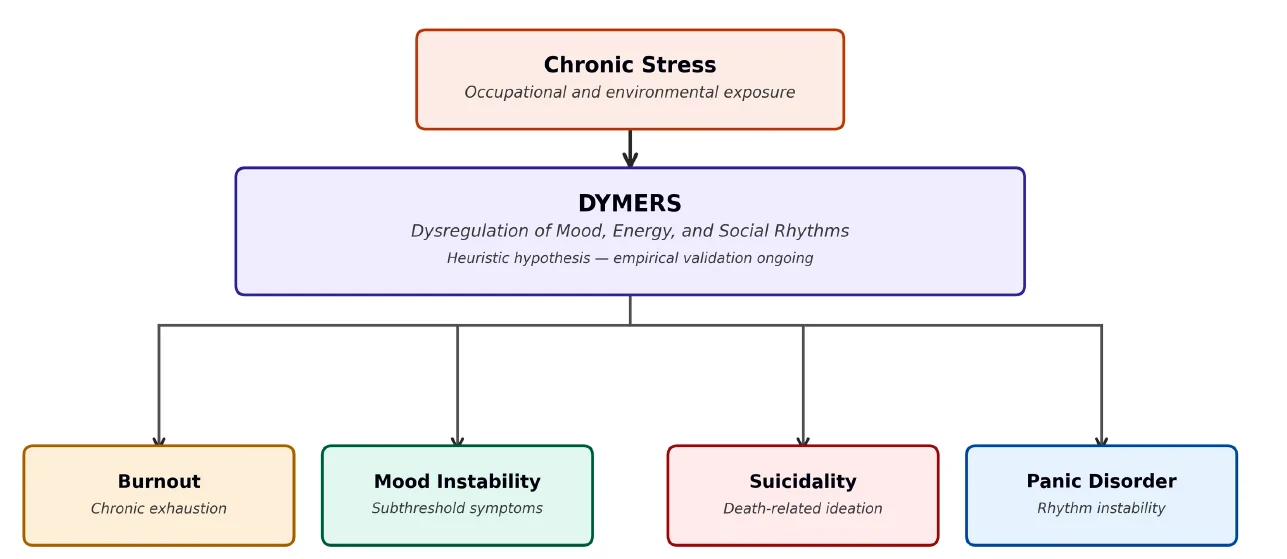
Conceptual model of the DYMERS framework. Chronic stress is proposed to contribute to dysregulation of mood, energy, and social rhythms (DYMERS), which may underlie a range of clinical outcomes including burnout, mood instability, suicidality, and panic disorder. Arrows represent proposed pathways based on preliminary evidence; causal relationships have not been established.

## LIST OF ABBREVIATIONS

HCWs: Healthcare Workers
DYMERS: Dysregulation of Mood, Energy, and Social Rhythms Syndrome
MDQ: Mood Disorder Questionnaire
COVID-19: Coronavirus Disease 2019
